# Assessment of Physicochemical Properties of Rituximab Related to Its Immunomodulatory Activity

**DOI:** 10.1155/2015/910763

**Published:** 2015-04-20

**Authors:** Mariana P. Miranda-Hernández, Carlos A. López-Morales, Nancy D. Ramírez-Ibáñez, Nelly Piña-Lara, Nestor O. Pérez, Aarón Molina-Pérez, Jorge Revilla-Beltri, Luis F. Flores-Ortiz, Emilio Medina-Rivero

**Affiliations:** ^1^Unidad de Investigación y Desarrollo, Probiomed S.A. de C.V., Cruce de carreteras Acatzingo-Zumpahuacán, 52400 Tenancingo de Degollado, MEX, Mexico; ^2^Dirección Médica, Probiomed S.A. de C.V., Avenida Ejército Nacional No. 499, Colonia Granada, Delegación Miguel Hidalgo, 11520 México, DF, Mexico

## Abstract

Rituximab is a chimeric monoclonal antibody employed for the treatment of CD20-positive B-cell non-Hodgkin's lymphoma, chronic lymphocytic leukemia, rheumatoid arthritis, granulomatosis with polyangiitis and microscopic polyangiitis. It binds specifically to the CD20 antigen expressed on pre-B and consequently on mature B-lymphocytes of both normal and malignant cells, inhibiting their proliferation through apoptosis, CDC, and ADCC mechanisms. The immunomodulatory activity of rituximab is closely related to critical quality attributes that characterize its chemical composition and spatial configuration, which determine the recognition of CD20 and the binding to receptors or factors involved in its effector functions, while regulating the potential immunogenic response. Herein, we present a physicochemical and biological characterization followed by a pharmacodynamics and immunogenicity study to demonstrate comparability between two products containing rituximab. The physicochemical and biological characterization revealed that both products fit within the same response intervals exhibiting the same degree of variability. With regard to clinical response, both products depleted CD20+ B-cells until posttreatment recovery and no meaningful differences were found in their pharmacodynamic profiles. The evaluation of anti-chimeric antibodies did not show differential immunogenicity among products. Overall, these data confirm that similarity of critical quality attributes results in a comparable immunomodulatory activity.

## 1. Introduction

Rituximab is a chimeric monoclonal antibody (mAb) approved by the FDA on 1997 as single agent for the treatment of relapsed or refractory, low-grade or follicular CD20-positive B-cell non-Hodgkin's lymphoma (NHL) and later, in 2006, as a treatment in combination with cyclophosphamide, doxorubicin, vincristine, and prednisone (CHOP) or other anthracycline-based chemotherapy regimens for patients with diffuse large B-cell lymphoma (DLBCL). In both cases it increases the response rate, diminishes disease progression events, and augments patients survival [1–3].

The molecular weight of rituximab is 144,544 Da and is constituted of 1328 aa. As an IgG isotype 1/*kappa*, rituximab contains a conserved N-glycosylation site at Asn297 of both heavy chains and is occupied by biantennary glycan structures, while murine variable regions and human constant regions define its chimeric nature.

Rituximab mechanisms of action comprise the binding of its Fab domain to CD20+ B-lymphocytes for the induction of apoptosis, either directly or throughout the recruitment of immune effector functions by its Fc domain, thus mediating B-cell lysis through complement-dependent cytotoxicity mechanism (CDC), after binding to C1q, or antibody-dependent cellular cytotoxicity mechanism (ADCC) once is recognized by the Fc*γ* receptors (Fc*γ*Rs) of effector cells, including natural killers, granulocytes, and macrophages [4–6].

Besides, the current knowledge concerning monoclonal antibodies (mAb) permits us to correlate the immunomodulatory activity of a mAb to critical quality attributes (CQAs) that depict its chemical composition and spatial configuration. On this regard, rituximab CQAs are associated with the appropriate recognition of CD20+ B-cells and the achievement of effector functions. Nevertheless, rituximab is subject to posttranslational modifications that can be acquired during its lifecycle, which provides an inherent physicochemical heterogeneity that could impact on its functionality [7, 8]. Although this heterogeneity is expected to occur batch to batch, its variability breadth can be controlled during the manufacturing process; thus, an acceptance range should be established for each CQA, depending on the observed safety and efficacy for the given process capabilities [9]. This is particularly important for the development of follow-on products, for which the demonstration of highly similar CQAs variability, along with the demonstration of comparable pharmacological responses with respect to the reference product, grant the biosimilar denomination [10–12].

Charge and glycosylation heterogeneities are relevant modifications that influence the immunomodulatory activity of mAbs. It is reported that acidic and basic isoforms, coming mainly from oxidation, deamidation, isomerization, amination, cyclization, glycation, and the presence of C-terminal lysines [13], could alter the mAb affinity to target and receptor molecules due to the modification of electrostatic and hydrophobic interactions with cell membranes. On the other hand, glycosylation contributes in maintaining stability of the mAbs' three-dimensional structure and modulates the binding interaction of the Fc domain to the effector cells, influencing CDC and ADCC mechanisms [14].

Regarding its immunogenicity, rituximab is considered as a low risk molecule although potentially immunogenic, since it does not exhibit cross-reactions with endogenous antibodies or autoimmunity induction; however, due to its chimeric nature, the production of human anti-chimeric antibodies (HACAs) may lead to the loss of efficacy in certain cases. Consequently, to discard any differential immunogenic response of a biosimilar rituximab, the comparability of its chemical composition (i.e., sequence and posttranslational modifications) should be demonstrated [15, 16]. Aggregation is another attribute that has been also identified as a CQA that participates in the development of an immunogenic response [17].

In this work, we conducted a comprehensive characterization followed up by a pharmacodynamics-immunogenicity clinical study of two products containing rituximab. The characterization exercise is focused on the comparison between the CQAs associated with the pharmacodynamic profile (PD) and the potential immunogenicity of rituximab such as protein identity (amino acid sequence), charge and glycosylation heterogeneity, aggregates content, and binding affinity to Fc*γ*RIIa and Fc*γ*RIIIa, while the biological characterization included measurement of the affinity to CD20 and potency through ADCC and CDC. The clinical evaluation was intended to demonstrate that both products exhibit the same behaviour as the result of a high physicochemical comparability.

## 2. Materials and Methods

### 2.1. Materials

Dibasic sodium phosphate heptahydrate (Na_2_HPO_4_·7H_2_O), monobasic sodium phosphate monohydrate (NaH_2_PO_4_·H_2_O), sodium chloride (NaCl), Tris-hydrochloride (NH_2_C(C_2_OH)_3_·HCl), and sodium hydroxide (NaOH) were obtained from J. T. Baker (Center Valley, PA). Sodium azide (NaN_3_), ammonium formate (CH_5_NO_2_), RPMI-1640 medium, fetal bovine serum (FBS), and formic acid were acquired from Sigma-Aldrich (St. Louis, MO). 2-Aminobenzamide (2-AB) was obtained from ProZyme Inc. (Hayward, CA); PNGase F was purchased from New England Biolabs (Woburn, MA) and Human IgG-Fc antibody from Bethyl Laboratories Inc. (Montgomery, TX). Tetramethylbenzidine (TMB) substrate was obtained from Thermo Scientific (Waltham, MA). ADCC Reporter Bioassay Kit and CellTiter 96 MTT were purchased from Promega (Madison, WI). Water was obtained from a Millipore Milli-Q Biocel system (Billerica, MA). All solutions were filtered through 0.2 *μ*m prior to analysis. Two products containing rituximab were employed: Kikuzubam from Probiomed S.A. de C.V., Mexico, and MabThera from F. Hoffmann-La Roche Ltd. Basel, Switzerland, as the reference product.

### 2.2. Mass Spectrometry (MS)

MS analyses were performed on a SYNAPT G2 HDMS (Waters Corp.; Manchester, UK) coupled to an ACQUITY UPLC H-Class Bio System (Waters Corp., Milford, MA) using an ESI source. Data was analyzed using BiopharmaLynx software (Waters Corp., Milford, MA) according to reported conditions [18].

### 2.3. Charge Heterogeneity

Capillary isoelectrofocusing (cIEF) was performed as we described in a previous report [19].

### 2.4. Glycosylation Heterogeneity

Glycan release and derivatization were performed as previously described [20]. Chromatographic separation was carried out using an ACQUITY UPLC H-Class Bio System with a linear gradient from 22 to 50% of acetonitrile using 100 mM ammonium formate aqueous solution at pH 4.50 as mobile phase A. Fluorescence detection was set at an excitation wavelength of 250 nm and 420 nm for emission, using a 150 × 2.1 mm, 1.7 *μ*m ACQUITY UPLC BEH glycan column coupled with a 1.7 *μ*m VanGuard BEH Glycan Precolumn from Waters Corp. (Milford, MA).

### 2.5. Aggregates

Rituximab purity was assessed on a 4.6 mm × 300 mm ACQUITY Ethylene Bridged Hybrid 200 analytical column with particle and pore diameters of 1.7 *μ*m and 200 Å, respectively (Waters Corp., Milford, MA). 20 mM phosphate buffer containing 150 mM NaCl and 3 mM NaN_3_ at pH 6.8 was used as mobile phase with isocratic gradient. UV detector was set at 280 nm in an ACQUITY UPLC H-Class Bio System.

### 2.6. Affinity Constants

Affinity constants under equilibrium (*K*_*a*_) were obtained by isothermal titration calorimetry (ITC) using a Nano ITC instrument from TA Instruments Inc. (New Castle, DE). 300 *μ*L of Fc*γ*RIIa and Fc*γ*RIIIa solutions at 5.0 *μ*M in PBS at pH 7.0 was titrated with continuous injections of 1.9 *μ*L rituximab solutions at 50 *μ*M in PBS at pH 7.0 until saturation at 25°C. NanoAnalyze Software v2.4.1 (TA Instruments Inc.; New Castle, DE) was used for the integration of heat signals and nonlinear regression analysis of the data.

### 2.7. Affinity to CD20

WIL2-S cell line (ATCC: CRL-8885) that expresses the CD20 antigen was incubated in the presence of different concentrations of rituximab in RPMI-1640 medium with 10% FBS for 2 h at 37°C. A secondary antibody (anti-human IgG-Fc) coupled to a radish peroxidase was added to detect the rituximab-WIL2-S complex after 1 h of incubation at 37°C, using TMB as substrate for 30 min at room temperature. Absorption was acquired at 450 nm. The test results were expressed as the relative percentage of the EC_50_ from the concentration-response curve of Kikuzubam with respect to the reference product.

### 2.8. CDC Assay

CD20 positive cells (WIL2-S, ATCC CRL-8885) were incubated in RPMI 1640 media with 10% of FBS with different concentrations of rituximab and complement human serum for 4 h at 37°C and 5% CO_2_. Then MTS substrate was added to each well with a further incubation of 2 h at the same conditions. The result of the assay was expressed as % relative potency, which is obtained comparing to the EC_50_ of the dose-response curve of Kikuzubam with respect of the EC_50_ of the dose-response curve of the reference product.

### 2.9. ADCC Assay

The ADCC Reporter Bioassay Kit from Promega (Madison, WI) was used according to manufacturer instructions. CD20 positive cells (WIL2-S, ATCC CRL-8885) were incubated with different concentrations of test antibody and a specific concentration of Jurkat transformed cells expressing CD16. Then a luminescent substrate was added with further incubation of 20 min. The result of the assay was expressed as % of relative potency of Kikuzubam with respect to the reference product.

### 2.10. Clinical Assessment

A double-blind, randomized, three-arm, and prospective study was designed. Two arms (1 and 2) were crossed after three cycles of treatment in order to review the expected use conditions of Kikuzubam and the possible impact on its efficacy as suggested by the Mexican health authorities.

The study protocol was approved by the IRB/IEC (Institutional Review Board/Independent Committee) of the participating research centres and by the Mexican health authorities (study protocol codes CAS/OR/01/CMN/083300410^a^1444-0114/2009 and CAS/OR/01/CMN/07330021830339-0816/2008). The study was conducted in accordance with the regulations and ethical principles based on the Declaration of Helsinki, the principles of the International Conference on Harmonization (ICH), and the Guidelines for Good Clinical Practice (GCP). An informed consent was obtained from all patients prior to their participation in the study. All procedures were explained in detail to the patients and all doubts were resolved.

The aim of the study was to evaluate the biological effects and safety of Kikuzubam compared to the reference product during six treatment cycles with CHOP therapy. Patients received either Kikuzubam or the reference product in each cycle, according to their treatment group, at a dose of 375 mg/m^2^ every 14 days by IV infusion. 59 patients diagnosed with moderate to high degree diffuse CD20+ B-cell non-Hodgkin lymphoma were randomly assigned into three groups. Group 1 was treated with Kikuzubam during the first three cycles and subsequently with the reference product for the remaining three cycles. Group 2 was initially treated with the reference product for three cycles and then with Kikuzubam for the next three cycles. Group 3 was treated with Kikuzubam throughout six cycles. All patients received concomitant CHOP chemotherapy for the six cycles. A 12-month observational period was included after the completion of the treatments.

Blood samples were collected from all patients for the determination of CD20+ B-cells levels as the PD endpoint on visits 1, 3, 4, 5, 6, 7, 8, 9, 10, 11, and 12 using a CD20 Becton Dickinson FITC Labelling Kit in a EPIC XL Beckman Coulter Inc. (Brea, CA) flow cytometer. Additionally, levels of serum human anti-chimeric antibodies (HACAs) were determined using the Human Antirituximab (HADA/HACA/HAMA/HAHA) IgG ELISA Kit for Human from Alpha Diagnostics (San Antonio, TX). The assay precision was determined from the graphs obtained with serum samples, resulting in a coefficient of variation (CV) lower than 10% with accuracy ranging from 90 to 110%.

### 2.11. Statistical Analysis

Analysis of covariance was performed to evaluate the effect of both treatments (Kikuzubam and the reference product) on the number of CD20+ B-cells relative to basal values (covariable). The ANOVA test was evaluated with a significance level of 0.05.

To avoid the effect of crossing treatments, the CD20+ B-cells depletion analyses were performed considering only the results from the first three cycles of treatment with either Kikuzubam or the reference product, in order to compare the response between treatments in a parallel design.

## 3. Results and Discussion

The physicochemical properties of rituximab are discussed according to its impact on PD and immunogenicity potential. Identity, heterogeneity, purity, and biological activity CQAs were studied by comparing several batches of Kikuzubam and the reference product.

### 3.1. Physicochemical Analyses

**Figure 1:**
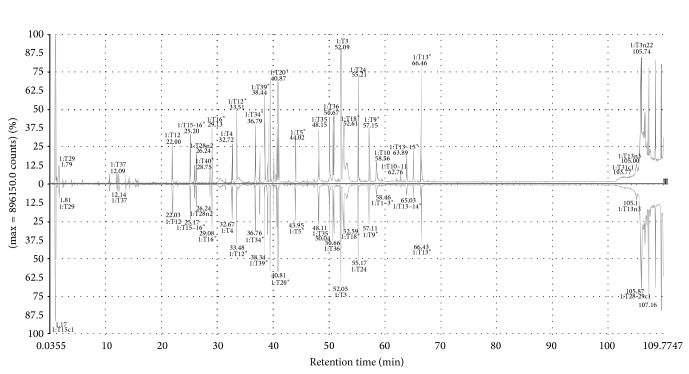
Chromatographic profiles of tryptic peptide mappings followed by MS/MS analyses of Kikuzubam (up) and the reference product (down).

The identity of both products was verified by its tryptic peptide chromatographic profiles followed by MS/MS analyses matched with the theoretical sequence of rituximab (Figure 1).

The theoretical sequence was obtained by reverse engineering, comprising a* de novo* protein sequencing of the reference product by ESI-MS/MS and MALDI PSD using trypsin, Glu-C, or Asn-N digestions along with EDMAN's degradation of selected fragments. This sequence was employed for the design and construction of the expression system of Kikuzubam (data not shown) that revealed inconsistencies in the invention patents [21, 22] of rituximab at the amino acid positions 14 and 219 of the heavy chain. Our results agree with the sequence published by other groups [23, 24] and the United States Pharmacopeia [25].

**Figure 2:**
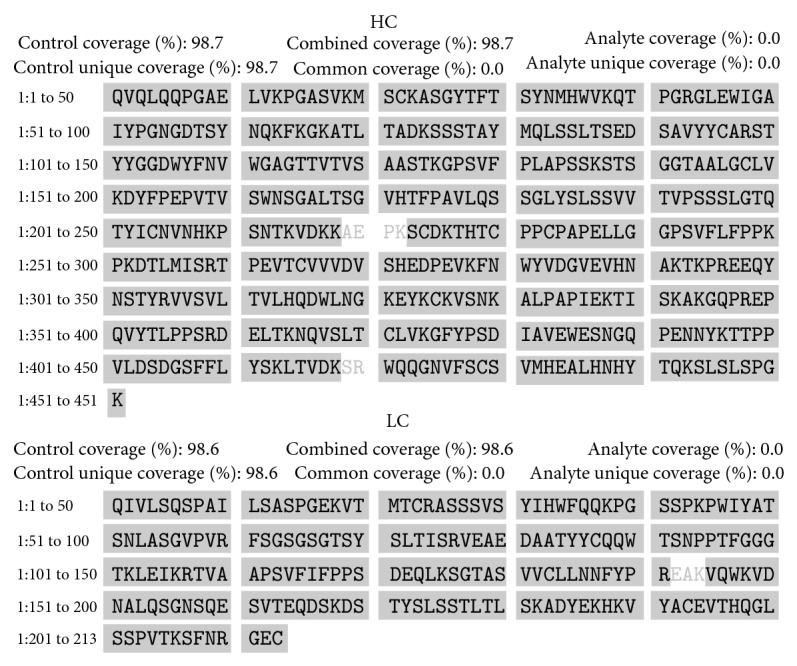
Sequence coverage of the heavy and light chain of Kikuzubam.

**Figure 3:**
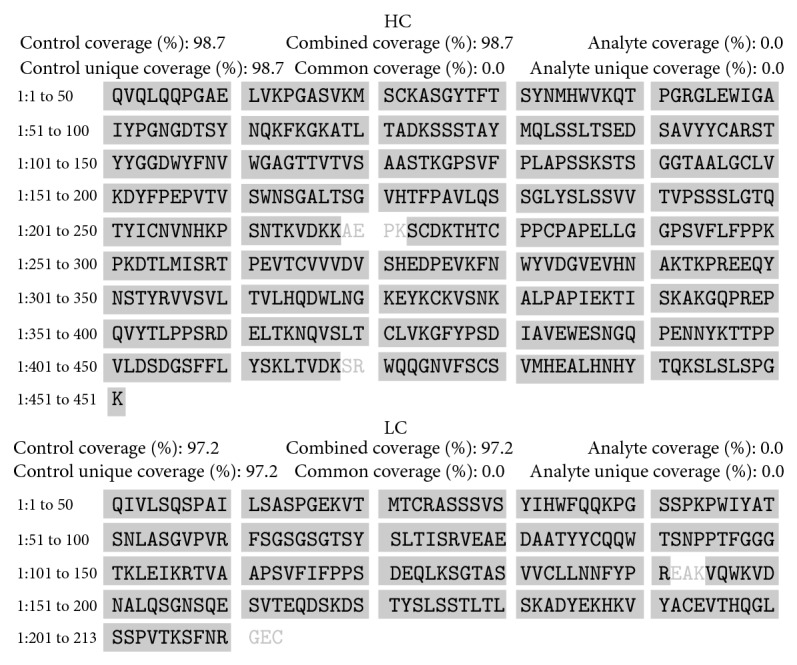
Sequence coverage of the heavy and light chain of the reference product.

For both products sequence verification, expressed as MS/MS sequence coverage, exceeded the accepted consensus value of 90%, being 98.7% and 98.6% for Kikuzubam and 98.7% and 97.2% for the reference product of their heavy and light chains, respectively (Figures 2 and 3).

In order to confirm the identity of Kikuzubam, exact mass of the whole deglycosylated molecule, coming uniquely from the amino acid sequence, was determined (Table 1). On the other hand, as we previously reported [18], correspondence between each glycoform and the theoretical mass (99.98%) was observed within and among Kikuzubam and the reference product. These results confirm that the primary sequences of both products are identical and also reveal that charge and glycosylation heterogeneities are comparable; thus, the risk of a differential immunomodulatory response is diminished.

The glycosylation heterogeneity of Kikuzubam and the reference product was also evaluated as a relevant CQA on the immunomodulatory activity of rituximab.  Table 2 shows the content of highly mannosylated, hybrid, sialylated, afucosylated and galactosylated glycoforms of both products. It is reported that these glycan isoforms could affect the affinity to the receptors involved in the effector function and stability of a mAb, due to charge and steric hindrances [26]. For instance, hybrid (bisected) and afucosylated glycans tend to increase the affinity to Fc gamma RIIIa, resulting in an enhanced ADCC response [27, 28], while sialylated isoforms could increase immune responses [14].

Nonetheless, the glycan heterogeneity of a biosimilar must correspond to the reference product. In this analysis, both products revealed similar glycan heterogeneity, which is consistent with the presence of the same glycoforms observed by the MS analyses of the whole molecule [18]. Although minor differences were found in the nonfucosylated and hybrid glycan content between products, no impact was observed on the potency or the efficacy of the ADCC assay afterwards (Figure 5) [14, 27, 28].

Regarding charge heterogeneity, changes higher than 1.0 units in the isoelectric point (pI) of a mAb could affect its therapeutic activity [13, 29], with the common pI variation observed during manufacturing being from 0.1 to 0.2 pI units the common pI variation observed during manufacturing [30]. On Table 3, we show the pI range, main isoform, and overall calculated pI values of Kikuzubam and the reference product. The observed differences were lower than 0.1 pI units confirming comparability of charge heterogeneity among the products.

Another relevant CQA related to the immunomodulatory activity of rituximab is the aggregation level, which involves the irreversible interaction of two or more denatured protein molecules revealing new epitopes that could stimulate the immune system. A positive correlation between protein aggregation and immunogenicity has been reported for therapeutic proteins, as well as affectations on the biological activity, either directly or indirectly through the formation of neutralizing or binding antibodies. Thus, the evaluation of aggregates is an important component of the analytical comparability assessment of therapeutic proteins. The aggregates content of Kikuzubam was comparable to the reference product (Table 4), which in both cases complied with the pharmacopeial established limit [25].

### 3.2. Biological Characterization

In addition to the physicochemical analyses, an extensive biological characterization to assess comparability of the functions (mechanisms of action) described for the reference product and Kikuzubam was performed through* in vitro* assays. These studies were designed taking into account the interactions of the Fab and Fc domains and their associated biological activities described in the literature (affinity to CD20, Fc*γ*RIIa, and Fc*γ*RIIIa).

**Figure 4:**
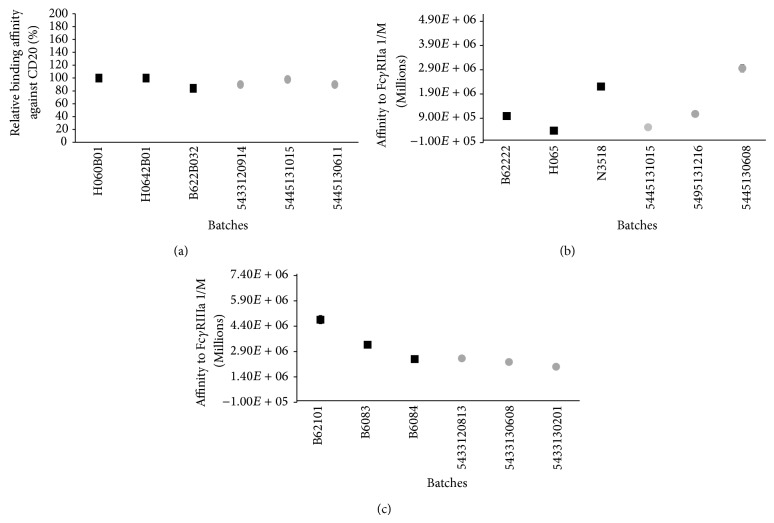
(a) Relative binding affinity against CD20, (b) affinity constants towards Fc*γ*RIIa, and (c) Fc*γ*RIIIa. Square marks represent the batches evaluated of reference product and circle marks represent the evaluated batches of Kikuzubam.

The main mechanism of action of rituximab is binding to CD20 [4, 6] whose interaction affinity is related to the structure of complementary domain regions (CDRs) of the Fab fragment, and this reveals the presence of the appropriate chemical and structural properties of this fragment. Our results showed that Kikuzubam and the reference product have comparable affinities to CD20 (Figure 4).

Rituximab also can induce the death of CD20+ B-cells by activating effector cells such as natural killer cells (NK), monocytes, and macrophages through the binding of the Fc*γ*R receptors to its Fc domain [31]. Clinical studies have shown that the affinity to Fc*γ*RIIa and Fc*γ*RIIIa receptors is associated with a better response to rituximab in patients with follicular lymphoma [32]. ITC results of affinity to Fc*γ*RIIa and Fc*γ*RIIIa of Kikuzubam and the reference product were within the same order of magnitude (Figure 4); thus, the modulatory functions that lead to B-cell depletion in both products are assumed to follow the same molecular basis.

**Figure 5:**
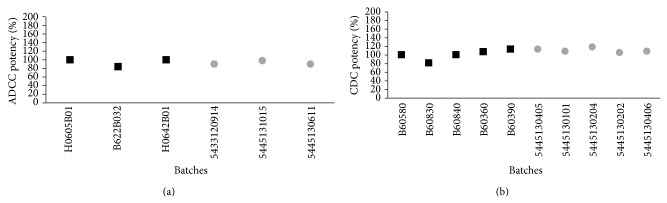
(a) ADCC and (b) CDC* in vitro* potency assays. Square marks represent the batches evaluated of reference product and circle marks represent the evaluated batches of Kikuzubam.

The described Fc and Fab affinities further modulate CDC and ADCC mechanisms of rituximab [5, 6] and both were evaluated comparatively for Kikuzubam against the reference product (Figure 5). These analyses also confirmed that the physicochemical characteristics of the Fc domain of Kikuzubam are capable of achieving the same biological functions with comparable potency as the reference product.

### 3.3. Pharmacodynamics

An abbreviated study conducted on CD20+ non-Hodgkin's lymphoma patients was designed to confirm that the physicochemical and functional characteristics of Kikuzubam are adequate to exhibit the same PD profile as the reference product.

**Figure 6:**
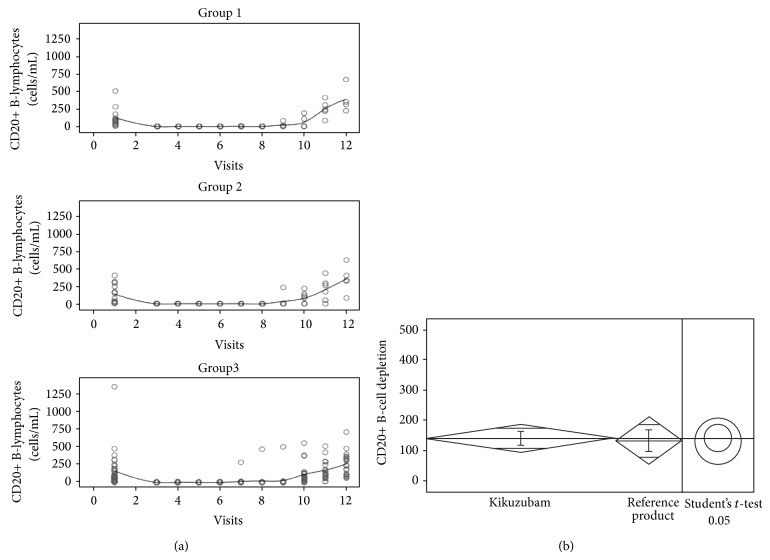
(a) Serum concentrations of CD20+ B-Lymphocytes measured along the PD study from patients of all groups. (b) Comparison of mean serum concentrations of CD20+ B-lymphocytes from all patients treated with Kikuzubam against the reference product.

CD20 was used as the main endpoint. During the treatment, CD20 B-cells were depleted to serum levels lower than 20 cell/mL in the three arms of the study (Figure 6). This is explained by the effect of both rituximab products since the levels of other blood components were recovered within 7 days after the completion of concomitant CHOP chemotherapy regimen and the application of granulocyte colony stimulating factor (Filgrastim, G-CSF). CD20 was the only component with no recovery in serum, despite the stimulation after the completion of chemotherapy. Once the six cycles of rituximab-CHOP were completed, a recovery in the serum levels of CD20+ B-cells for the three groups of the study was observed. Nonmalignant recovery was demonstrated by PET images as the absence of neoplasms (data not shown).

In order to determine the comparability of the primary endpoint, statistical analyses for the three arms before crossing were performed. Shapiro-Wilk test revealed departures from normality of the data (*P* < 0.05). However, homoscedasticity was demonstrated through a Levene test (*P* > 0.05). Mean comparison among the groups was performed by Student's *t*-test and Wilcoxon tests, revealing no significant differences in CD20+ depletion between Kikuzubam and the reference product (*P* > 0.05) (Table 5). Combined groups were also analysed using data from arms 1 and 3 to compare all patients treated with Kikuzubam against the reference product, one outlier was excluded. The results obtained from this exercise also revealed no meaningful differences among treatments (Figure 6).

### 3.4. Immunogenicity

The production of antichimeric human antibodies (HACAs) as a result of the loss of tolerance to rituximab by the immune system was evaluated on the three study arms.

On arm 1, two patients showed positive results for the screening test of HACAs right after the shift from Kikuzubam to the reference product on visit 5 (Table 6). Thus, the immunogenic response had to be triggered before the medication shifting, since the first humoral immunogenic response is the production of IgM antibodies, which half-life in plasma is approximately four weeks, followed by isotype switching to IgG, if loss of tolerance with the consequent HACAs production is presented. Also, on arm 2, the presence of HACAs in two patients was detected before the shift of treatment (Table 6). Then the immunogenic response produced in 4 out of 28 patients from the first two groups cannot be considered as a consequence of the drug shifting. The immunogenic response was analogous between Kikuzubam and reference product; therefore, no differential immunogenicity was observed.

Likewise, three patients from arm 3 presented positive results for the screening HACAs test (Table 6). These data suggest that the proportion of patients positive to HACAs was comparable between all study arms. The presence of these antibodies did not represent a risk to the patient safety and did not justify abandoning the study. Overall, the biological effect was comparable to HACAs-negative patients between both products.

The hematologic recovery after R-CHOP cycles, even in patients positive to HACAs screening test for both Kikuzubam and the reference product, was accomplished within the expected period reported in studies with chemotherapy; thus, it can be inferred that neither the HACAs developed by Kikuzubam nor the reference product had a negative effect on the hematologic recovery of patients included in the study.

## 4. Conclusions

The comprehensive physicochemical, biological, and* in vitro* characterization studies, including the verification of amino acid sequence, glycosylation and charge heterogeneity, aggregates content, and affinity to CD20, Fc*γ*IIa, and Fc*γ*IIIa receptors, provided valuable information to demonstrate comparability between Kikuzubam and the reference product. The information provided by these analyses supported the design of a rational clinical evaluation to demonstrate similar immunomodulatory response through the pharmacodynamics and immunogenicity profiles. Physicochemical along with biological comparability resulted in a similar immunomodulatory activity between the evaluated products.

## Figures and Tables

**Table 1:** Analysis of the exact mass of Kikuzubam and the reference product.

Product	Batch	Experimental mass (Da)
MabThera	B60480	144190.99
	B60711	144190.04
	B6084	144190.96

Kikuzubam	RPPP11003	144191.29
	RPPP11014	144191.40
	RPPP12015	144191.93

**Table 2:** Glycosylation microheterogeneity obtained by HILI-UPLC. Variation is presented as confidence interval at 95% (*n* = 3).

Product	Batch	Nonfucosylated (%)	Hybrid (%)	Sialylated (%)	Galactosylated (%)	High mannose (%)
MabThera	H0605	1.81 ± 0.04	5.00 ± 0.32	0.72 ± 0.08	43.34 ± 1.42	4.37 ± 0.42
	N3518	1.69 ± 0.11	3.48 ± 0.24	0.98 ± 0.22	46.21 ± 1.54	3.19 ± 0.54
	B62222	1.89 ± 0.11	2.91 ± 0.18	0.82 ± 0.12	45.58 ± 0.47	3.03 ± 0.17

Kikuzubam	5445130608	0.65 ± 0.21	2.72 ± 0.39	0.80 ± 0.21	57.08 ± 8.52	3.25 ± 0.43
	5445131216	0.65 ± 0.21	2.80 ± 0.08	0.71 ± 0.13	56.35 ± 0.64	3.31 ± 0.16
	5445131015	0.67 ± 0.05	3.19 ± 0.25	0.77 ± 0.13	54.26 ± 3.02	3.51 ± 0.23

**Table 3:** Isoelectric point by cIEF. Variation is presented as confidence interval at 95% (*n* = 3).

Product	Batch	Main isoform (pI units)	Most acidic variant (pI units)	Most basic variant (pI units)	Global pI (pI units)
MabThera	M0605	9.31 ± 0.00	8.68 ± 0.00	9.49 ± 0.00	9.07 ± 0.06
	N3518	9.31 ± 0.00	8.68 ± 0.00	9.49 ± 0.00	9.09 ± 0.06
	B62222	9.31 ± 0.00	8.68 ± 0.00	9.49 ± 0.00	9.09 ± 0.06

Kikuzubam	5445130608	9.30 ± 0.00	8.64 ± 0.00	9.42 ± 0.00	9.03 ± 0.07
	5445131015	9.30 ± 0.01	8.64 ± 0.01	9.42 ± 0.00	9.02 ± 0.08
	5445131216	9.29 ± 0.01	8.63 ± 0.01	9.42 ± 0.00	9.01 ± 0.08

**Table 4:** Aggregates content obtained by SE-UPLC. Variation is presented as confidence interval at 95% (*n* = 3).

Product	Batch	Aggregates (%)
MabThera	B62222	0.11 ± 0.01
	H0605	0.07 ± 0.01
	N3518	0.09 ± 0.02

Kikuzubam	5445131015	0.13 ± 0.03
	5445130608	0.10 ± 0.05
	5445131216	0.25 ± 0.03

**Table 5:** Comparability of the primary endpoint between treatments.

Patients *n*	Arms	Student's *t*-test *P*	Wilcoxon *P*
10 versus 13	1 versus 2	0.5114	0.5558
38 versus 13	1 and 3 versus 2	0.5742	0.6421
37 versus 13	1 and 3^∗^ versus 2	0.8603	0.7401

^∗^Exclusion of one outliner.

**Table 6:** HACAs determination in the immunogenicity study.

Arm	Number of patients	Positive HACAs patients	Positive HACAs patients (%)
1	15	2	13
2	13	2	15
3	31	3	10
